# Potential applications of ultrasound-based leadless endocardial pacing in adult congenital heart disease

**DOI:** 10.1016/j.hrthm.2024.09.006

**Published:** 2025-02

**Authors:** Nadeev Wijesuriya, Felicity De Vere, Sandra Howell, Nilanka Mannakkara, Paolo Bosco, Alessandra Frigiola, Seshandri Balaji, Henry Chubb, Steven A. Niederer, Christopher A. Rinaldi

**Affiliations:** 1 Department of Biomedical Engineering and Imaging Sciences, King’s College London, London, United Kingdom; 2 Department of Cardiology, Guy’s and St Thomas’ NHS Foundation Trust, London, United Kingdom; 3 Oregon Health and Science University, Portland, Oregon; 4 Stanford University, Stanford, California; 5 National Heart and Lunk Institute, Imperial College London, London, United Kingdom; 6 Alan Turing Institute, London, United Kingdom

**Keywords:** Leadless pacing, Congenital heart disease, Cardiac resynchronization therapy, Pacing-induced cardiomyopathy, Heart failure

## Introduction

Cardiac device therapy is frequently required for individuals with adult congenital heart disease (ACHD), whether it is for bradyarrhythmia, ventricular tachyarrhythmia, or cardiac resynchronization therapy (CRT). Compared with a standard adult population, there are special considerations in planning device implantation in ACHD patients, especially those with complex anatomy. These include issues with access due to native or postsurgical anatomic variations, increased likelihood of multiple interventions during a patient’s lifetime, and challenges in pacing a single-ventricle (SV) circulation.

This article examines the potential applications of leadless ultrasound-based endocardial pacing using the WiSE-CRT System^1^ (EBR Systems Inc, Sunnyvale, CA) in the ACHD population. We first outline the indications for pacing and CRT in ACHD and the challenges of lead-based systems. Next, we describe the current experience of leadless pacing in ACHD. We then provide an overview of the WiSE-CRT system and the contemporary evidence for its use. Following this, we suggest where the system has the potential for benefit in the ACHD population. Finally, we discuss the current limitations of the WiSE-CRT device and how future technologic advancements may enhance its suitability for this cohort of complex patients.

## Pacing indications in ACHD

A summary of the guidelines for pacing in ACHD is shown in Table 1, with an outline of the incidence of sinoatrial node (SA) dysfunction and atrioventricular (AV) block for different complex lesions shown in Table 2.^2^ The substrate for device implantation is frequent in this population, both through associated congenital conduction disease and by iatrogenic effect after surgery.

**Table 1:** Indications for pacing in adults with complex congenital heart disease

Class	Clinical indication	Level of evidence
I	Symptomatic sinus node dysfunction, including documented sinus bradycardia or chronotropic incompetence that is intrinsic or secondary to required drug therapy	C
	Symptomatic bradycardia in conjunction with any degree of AV block or with ventricular arrhythmias presumed to be because of AV block	B
	Postoperative high-grade second- or third-degree AV block that is not expected to resolve	C
IIa	Impaired hemodynamics, as assessed by noninvasive or invasive means, due to sinus bradycardia or loss of AV synchrony	C
	Sinus or junctional bradycardia for the prevention of recurrent IART	C
	Adults with complex CHD and an awake resting heart rate (sinus or junctional) <40 beats/min or ventricular pauses >3 seconds	C
IIb	Adults with CHD of moderate complexity and an awake resting heart rate (sinus or junctional) <40 beats/min or ventricular pauses >3 seconds	C
	History of transient postoperative complete AV block and residual bifascicular block	C
III	Pacing is not indicated in asymptomatic adults with CHD and bifascicular block with or without first-degree AV block in the absence of a history of transient complete AV block	C
	Endocardial leads are generally avoided in adults with CHD and intracardiac shunts	B

AV = atrioventricular; CHD = congenital heart disease; IART = intra-atrial reentrant tachycardia.

**Table 2:** Incidence of arrhythmia in complex congenital heart disease

Anatomy	Intervention	SA node dysfunction (%)	AV block (%)	Ventricular arrhythmias (%)
Tetralogy of Fallot	Surgical repair	1–2	5	2–15
D-TGA	Post-Mustard	60–82		2
	Post-Senning	22–35		
	All atrial switch		4	7
	Post–arterial switch	1	1–2	<1
ccTGA (L-TGA)	All	<5	20–25	1
Ebstein anomaly	All	Minimal data	4	1
Left atrial isomerism	All	19–80	16	Minimal data
Univentricular heart	All Fontan	5–27	Anatomy dependent	4

AV = atrioventricular; ccTGA = congenitally corrected transposition of the great arteries; D-TGA = transposition of the great arteries with d-looping (right-hand topology); L-TGA = transposition of the great arteries with l-looping (left-hand topology); SA = sinoatrial.

## Challenges in transvenous pacing in ACHD

**Figure 1:**
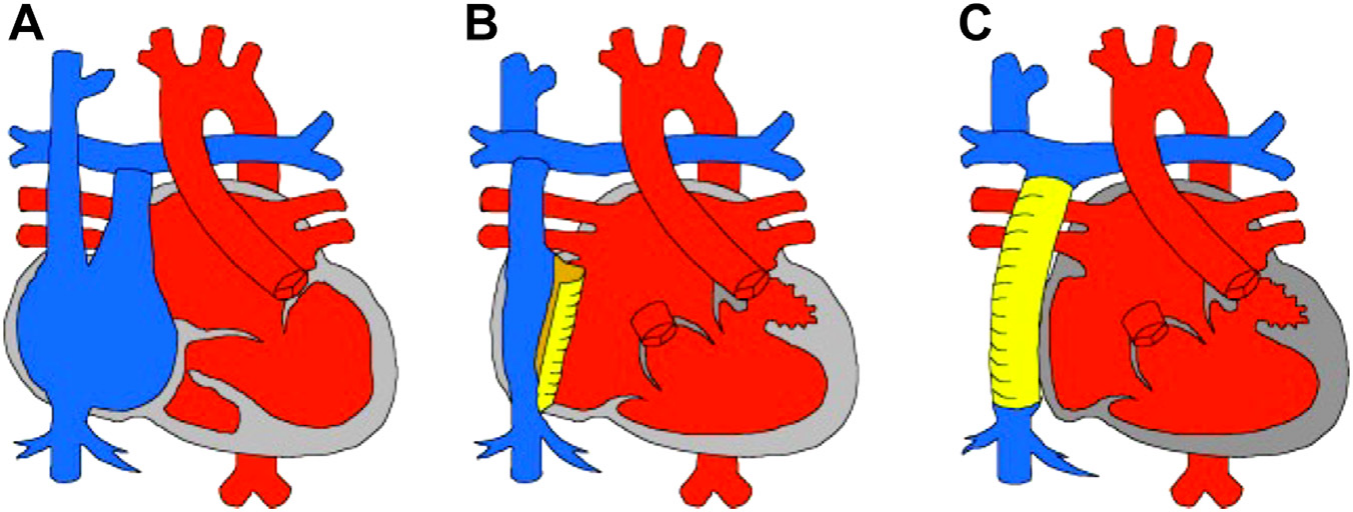
Type of Fontan procedures. **A:** Atriopulmonary connection (AP Fontan). **B:** Lateral tunnel. **C:** Extracardiac conduit. Reproduced from^4^, with permission.

Whereas transvenous devices are standard in conventional adult patients, implantation poses specific challenges in ACHD. In particular, those with complex anatomy present issues with regard to venous access, residual shunts, and SV circulations. The classic example of this would be those with a Fontan circulation (Figure 1).^4^

**Figure 2:**
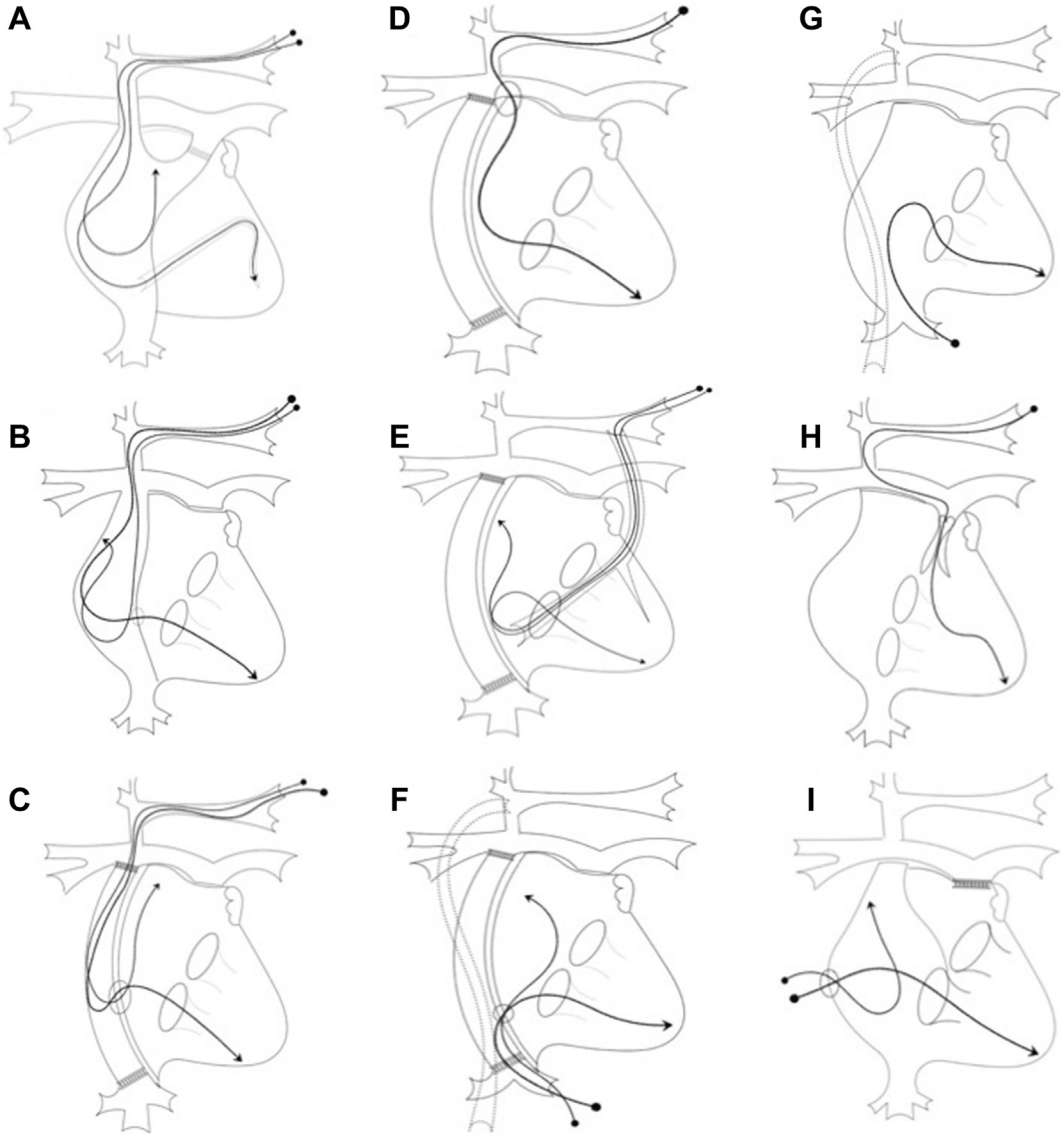
Graphic illustration of different methods of endocardial pacing. **A:** Atrial lead in right atrial appendage and ventricular lead through the coronary sinus in atriopulmonary Fontan. **B:** Atrial lead in lateral atrial wall and ventricular lead through fenestration in lateral tunnel Fontan. **C:** Both atrial and ventricular lead through fenestration in extracardiac conduit Fontan. **D:** Ventricular lead through a fenestration created between floor of right pulmonary artery and atrium with a Brockenbrough needle. **E:** Atrial and ventricular lead through venous collaterals after Fontan. **F:** Transhepatic leads through fenestration in extracardiac conduit Fontan following Kawashima shunt. **G:** Transhepatic lead through hepatic vein following total cavopulmonary Kawashima shunt. **H:** Ventricular lead through narrowed pulmonary valve in pulsatile Glenn shunt. **I:** Periatrial atrial and ventricular lead through a purse-string suture in right atrial wall. Reproduced from^6^, with permission.

Pacing is the most common intervention after Fontan surgery, with reported rates between 5% and 41% during 15 to 20 years.^5^ Although SN dysfunction is more common than AV block, ventricular pacing is still frequently required, especially in those receiving antiarrhythmic medication for atrial tachyarrhythmias. Complete heart block is also more common in certain morphologies of SV, such as in heterotaxy, common AV valves, and AV discordance.^6^ Several innovative techniques have been described to provide transvenous pacing in these patients (Figure 2),^6^ including baffle punctures, use of venous collaterals, and transhepatic pacing.

Whereas each technique will have advantages and challenges, in general there are common issues. The first is increased thromboembolic risk with leads in the systemic ventricle. Khairy and associates^7^ reported in a retrospective cohort of patients with right-left intracardiac shunts that transvenous leads were associated with a 2-fold increased thromboembolic risk. These findings were mirrored in the ALSYNC study,^8^ in which endocardial left ventricular (LV) pacing was associated with a 10.6% risk of stroke/transient ischemic attack despite anticoagulation. Of note, anticoagulation can be a particular problem in Fontan patients, who have a high prevalence of cirrhosis and coagulopathy.^9^

Second, whereas coronary sinus pacing may be possible in some lateral tunnel and atriopulmonary Fontans,^10^ transvenous ventricular pacing in Fontan patients will involve a lead crossing the systemic AV valve in most circumstances. Pacing leads are known to increase the risk of valvular regurgitation 2-fold,^11^ through fibrosis, leaflet perforation/impingement, or chordae disruption, which can have a significant impact on a physiologically vulnerable Fontan circulation.

Finally, there are unique challenges related to extraction when an unconventional implantation technique has been used, for example, baffle tears or leaks, systemic ventricular perforation, and thromboembolism, which may have even graver consequences in Fontan patients. In view of these inherent risks, complex ACHD patients currently almost always require alternative pacing options to transvenous leads.

## Epicardial pacing in ACHD

**Figure 3:**
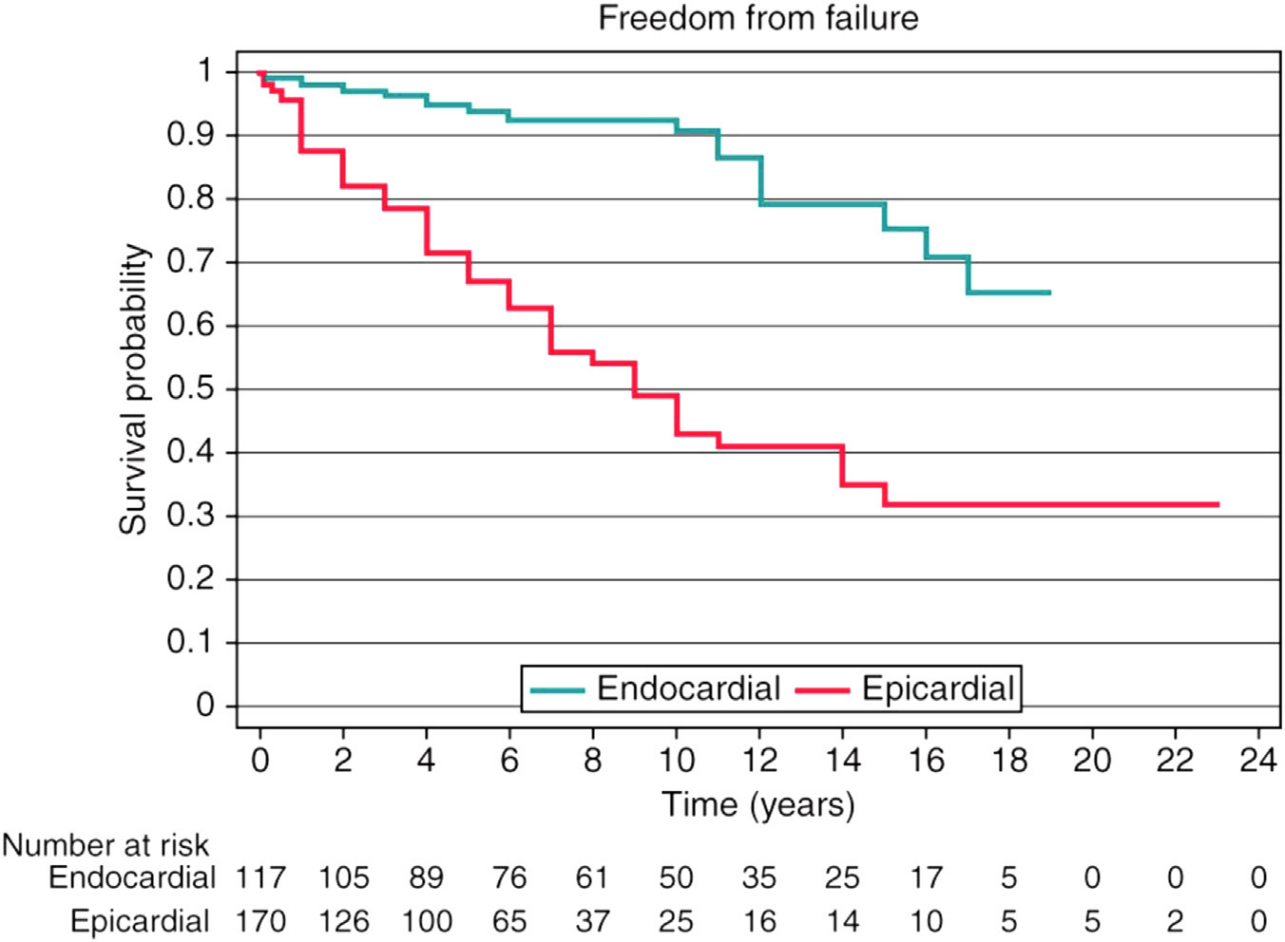
Survival analysis of epicardial vs endocardial pacing systems. Reproduced from^12^, with permission.

Epicardial pacing is common in ACHD, with a prevalence of >50% in those requiring ventricular pacing.^12^ It has several advantages over endocardial leads in this population: venous access is not needed; the leads can be placed concurrently during surgery; and thromboembolic and endocarditis risk is lower. Beyond patient-specific surgical implantation risk, the primary issue here is lead reliability. Multiple studies have demonstrated an increased failure rate with epicardial systems compared with endocardial.^12^, ^13^, ^14^ In a retrospective analysis of 287 ACHD patients, Silvetti and coworkers^12^ reported a 40% failure rate of epicardial systems compared with 13% with endocardial systems at median 5-year follow-up (Figure 3). This supported findings from McLeod and coworkers,^13^ who observed an overall lead or generator complication rate of 28% in 106 ACHD patients during 11-year follow-up. Epicardial systems were more likely to develop lead failure (*P* < .0001), most commonly in the ventricular lead (*P* < .0001). Regression analysis revealed an epicardial system to be an independent predictor of lead failure.

As well as lead reliability, another factor to consider is the long-term effect of surgical incisions. Although ventricular leads can be placed through thoracotomy or subxiphoid incision, atrial leads generally require full sternotomy. Longer term complications include detrimental effects on lung function and chronic pain syndromes.^15^ In addition, repeated sternotomy affects the risk of bleeding and death of future procedures.^16^ This is especially relevant in ACHD patients, in whom multiple interventions (device, valvular, transplant) may be required in a lifetime. Furthermore, there have been descriptions of coronary artery compression from epicardial leads, with 1 center reporting an incidence of 5.5% in a 145-patient cohort.^17^ As such, whereas epicardial systems are the standard of care in many forms of ACHD for the moment, newer pacing technologies may become more attractive in treating this high-risk and complex group of patients.

## Leadless pacing in ACHD

Leadless pacemakers have emerged as a reliable technology for right ventricular (RV) pacing in adult patients, with the Micra (Medtronic, Minneapolis, MN) currently being most commonly used.^18^ Leadless devices have also progressed in achieving AV synchrony, first with the Micra AV^19^ and now with the AVEIR atrial system (Abbott Cardiovascular, Plymouth, MN),^20^ although with the limitations of less reliable tracking at higher heart rates with the Micra, which is relevant particularly in younger patients. There is limited evidence of leadless pacing in ACHD. Shah and colleagues^21^ reported results of a pediatric registry of 63 patients, 20 of whom had CHD (tetralogy of Fallot, ventricular septal defect, atrial septal defect, congenitally corrected transposition of the great arteries). During mean follow-up of 9 months, there were 10 (16%) complications including 1 pericardial effusion, 1 femoral venous thrombus, and 1 retrieval due to high thresholds. Bassareo and Walsh^22^ have also reported their experience of Micra implantation in 15 ACHD patients, 3 with a systemic RV and 2 with SV physiology. No major complications were encountered at 6-month follow-up. Technical challenges have limited widespread uptake of Micra in SV patients; current delivery sheaths are generally too short for retrograde femoral artery access and too large bore to gain trans-baffle access. Cases of implantation in Fontan patients have primarily been through a transcarotid approach.^23^

Long-term management is the key issue with these leadless devices. Whereas battery longevity is generally good, roughly 10 years, ACHD patients will likely require several lifetime replacements. There is some evidence that the conventional right ventricle can accommodate up to 3 Micra systems^24^; however, this may not be sufficient in a younger population. In addition, computer modeling studies tracking the motion and collisions of LV intracardiac structures and leadless devices have suggested that devices such as Micra would need to be reduced in size by >40% to leave the LV endocardium collision free throughout the cardiac cycle and thus to avoid long-term effects on contractility.^25^ These issues may be lessened if leadless devices can be extracted safely when replacement is needed. Pertinent long-term data are awaited with interest, particularly in the case of AVEIR, which has design elements facilitating retrieval/extraction, making it potentially more attractive in a younger population.^26^

## CRT in ACHD

The primary application of CRT in conventional adult patients is to correct bundle branch block in those with severely impaired LV function. In these patients, CRT has abundant evidence demonstrating improvement in quality of life, cardiac function, and life expectancy.^27^^,^^28^ Conversely, in ACHD, CRT is mainly used to treat pacing-induced cardiomyopathy (PICM), commonly as a result of iatrogenic postsurgical AV block, or as a therapeutic adjunct in a failing SV.^29^ Chubb and coworkers^30^ reported, in an international study of 236 patients with an SV receiving ventricular pacing, a 3-fold higher rate of heart failure events compared with unpaced matched controls. In a retrospective study of 106 ACHD patients, Moore and coworkers^31^ observed a 47% rate of PICM in those with a ventricular pacing burden >70%. Patients with PICM were 3 times more likely to be admitted with heart failure (44% vs 15%; *P* = .002). CRT was performed in 11 patients, with 9 responders (82%). These findings support those of Dubin and coworkers,^32^ who reported that in a pediatric population (median implantation age, 12.8 years; 71% congenital heart disease), CRT produced a mean 13% increase in ejection fraction, with 3 patients previously on the heart transplant waiting list improving sufficiently to be removed from it. There have also been small case series in which epicardial CRT provides benefit in SV physiology. Cecchin and colleagues^33^ demonstrated that 8 of 13 patients with an SV exhibited a “strong CRT response,” defined as either an improvement in New York Heart Association class or increased ventricular function by ≥10%. A separate study noted that maximal pacing lead separation seemed to be beneficial when employing CRT in an SV.^34^

Whereas conventional CRT, that is, transvenous epicardial CRT to the coronary sinus branches, is possible in certain ACHD patients, this is prohibited in many by the postsurgical anatomy. For example, after an atrial switch for transposition of the great arteries with d-looping, both the coronary sinus and venous baffle may provide access to the subpulmonary LV, with no systemic RV access. Often, a surgical epicardial CRT approach is employed, and as described before, this entails its own challenges. Newer devices may have the potential to disrupt this field, in particular leadless technologies with the ability to pace the systemic ventricle.

## Leadless endocardial pacing of the systemic ventricle

**Figure 4:**
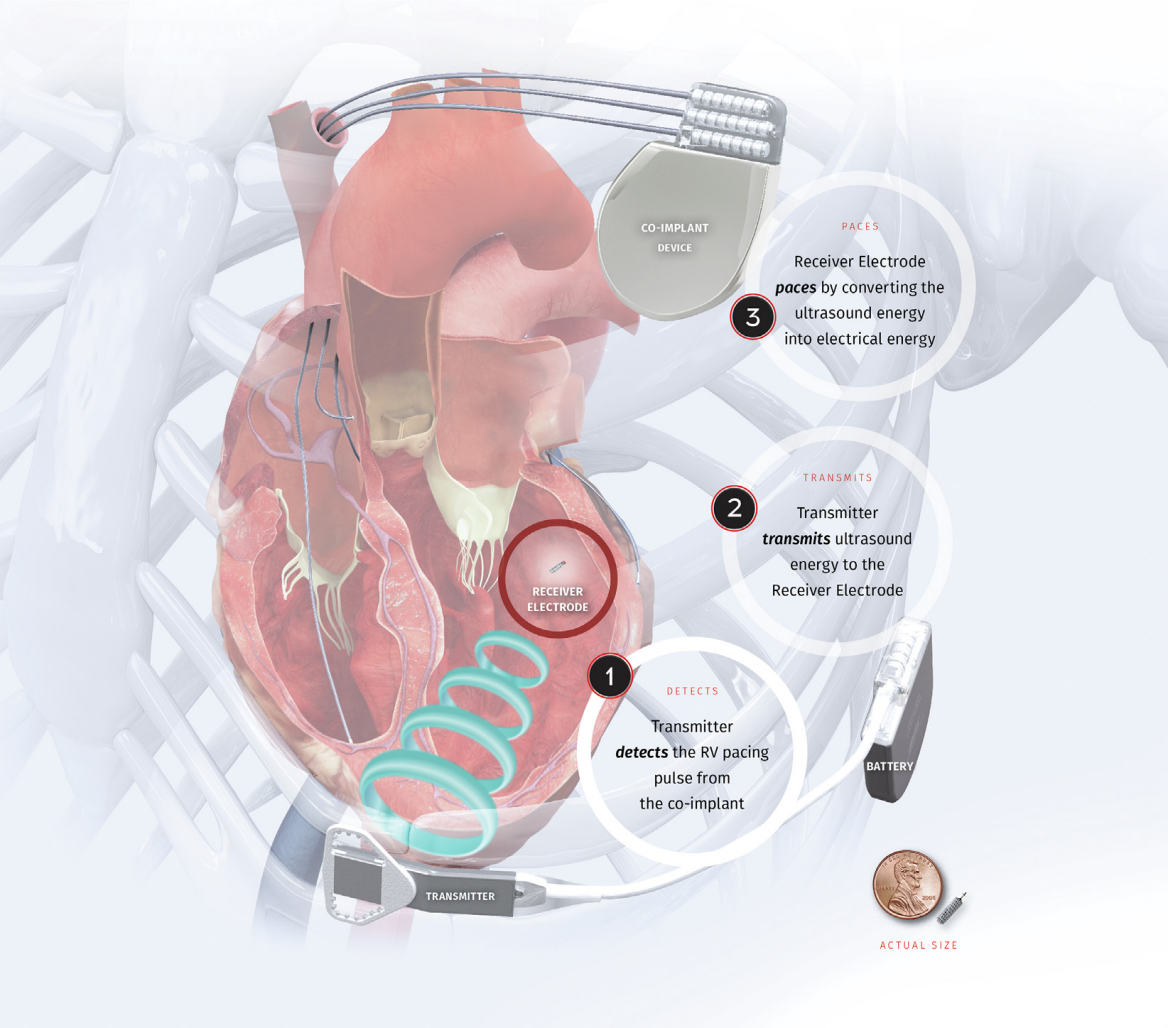
Components of the WiSE-CRT System. RV = right ventricular. Reproduced from^35^, with permission.

Leadless endocardial pacing of the systemic ventricle has the potential to be a valuable tool in the ACHD population. Currently, the WiSE-CRT system is the only known device that is specifically designed to pace the systemic ventricle, given its conventional use in LV pacing.^1^ The system consists of a battery connected to an ultrasound transmitter, implanted subcutaneously at the fourth, fifth, or sixth intercostal space, and the receiver electrode, implanted in the systemic ventricular cavity through femoral access (Figure 4).^35^ The system requires a “co-implant” in situ capable of producing continuous ventricular (conventionally RV) pacing, which can be a transvenous, leadless, or epicardial device. The transmitter and battery detect the pacing pulse emitted by the co-implant. Within 10 ms of detection of the pacing stimulus, the transmitter emits a number of ultrasound pulses to locate the receiver electrode. Once the transmitter is electronically optimally aligned, a longer ultrasound wave is emitted, which is detected and converted to a pacing stimulus by the receiver electrode. This results in systemic ventricular pacing and thereby nearly simultaneous biventricular pacing when used conventionally.

Leadless CRT has been demonstrated to be effective in observational and registry studies.^36^^,^^37^ A meta-analysis of 181 adult patients reported a mean increase in LV ejection fraction of 6.3%, with an echocardiographic response rate of 54%.^38^ The most recent trial was the pivotal SOLVE-CRT study.^39^ This was an international multicenter trial with a randomized phase (n = 108) and a subsequent single-arm phase (n = 75). The trial study met its primary efficacy end point, with a mean reduction in LV end-systolic volume of 16.4%. The primary safety end point was also met, with an 81% rate of freedom from type 1 complications. Of note, the primary follow-up in this study was 6 months, and long-term outcome data are still awaited.

There are several advantages of using the WiSE-CRT device in the ACHD population. It is designed to endocardially pace the systemic ventricle. The electrode is smaller (10 mm in length, 2.67 mm in diameter) than its RV pacing counterparts, enabling safe implantation without impeding systolic motion. Animal necropsy studies have shown that the receiver electrode is fully endothelialized within 90 days.^40^ In clinical practice, after 3 months of dual antiplatelet therapy, recipients are stepped down to a single antiplatelet agent, thus avoiding the need for lifelong anticoagulation if it is not otherwise indicated. Importantly, implantation is performed through the groin, by either retrograde arterial access or venous antegrade interatrial transseptal access,^41^ which provides optionality for patients with challenging anatomies. The receiver electrode can be implanted anywhere within the ventricular cavity, thus enabling the implanter to select the optimal pacing location on the basis of factors such as device thresholds, QRS narrowing, distance from the existing pacing leads, and avoidance of surgical scar. Recent advances^42^ have also enabled septal placement of the receiver electrode, with several reports of leadless left bundle area pacing being performed. Finally, the absence of an additional transvenous lead makes this a useful option in those with a higher endocarditis risk.

There are of course limitations with the current technology. The subcutaneous battery is large (49 × 111 × 12 mm, compared with approximately 55 × 60 × 6 mm for a CRT pacemaker), which poses an issue in younger patients. In addition, the battery longevity is less than that of transvenous devices (roughly 2–5 years), which subjects patients to multiple generator replacements and the potential complications of these. A smaller profile, rechargeable battery is in development and should alleviate this issue. In addition, whereas no significant longer term device complications, such as embolization or erosion, have been reported, ongoing follow-up data from currently implanted patients will be required to determine whether there are any such problems that would disproportionately affect younger patients.

One significant issue that requires addressing moving forward is the question of effective pacing percentage. Unlike a functional transvenous device, this ultrasound-based pacing method does not reliably produce a 100% rate of ventricular capture. The rate of capture reported in the real-world registry study was approximately 94%.^36^ Although this may be acceptable when WiSE-CRT is used in conjunction with the co-implant to deliver CRT, it would not be in situations in which it alone is relied on to deliver ventricular pacing in those with AV block. Improving this reliability will be crucial for the device to be used to deliver bradycardia-indicated pacing.

Related to this is the issue of standalone pacing. Currently, WiSE-CRT can only pace on detection of a pacing implant from a co-implanted device. This fits its primary use in delivery of biventricular pacing; however, more innovative methods need to be applied to deliver standalone ventricular pacing, for example, in patients with superior central venous occlusion. This could include timing leadless pacing from a subthreshold impulse generated by redundant epicardial leads or using an investigative feature of the WiSE-CRT programmer to deliver leadless pacing by timing from an atrial impulse at a fixed AV delay. Another as yet untested option would be timing the WiSE-CRT system to a subthreshold pacing pulse emitted by a *subcutaneously* placed co-implanted device. This would circumvent problems related to transvenous or thoracic access pertinent to the co-implant and as such would have universal applicability in this population. Animal studies would be required to test the feasibility of this concept.

**Figure 5:**
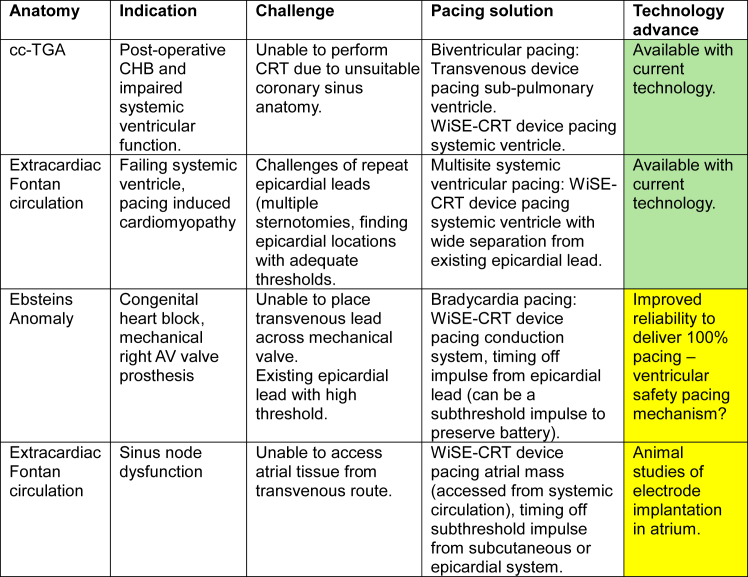
Examples of situations in which WiSE-CRT may be beneficial and the necessary technology advancement. AV = atrioventricular; ccTGA = congenitally corrected transposition of the great arteries; CHB = complete heart block; CRT = cardiac resynchronization therapy.

Figure 5 shows examples of potential patients in whom leadless endocardial pacing could be used, for varying indications, and the advances needed in current technology for this application to be feasible. Related to these indications, a particularly intriguing and attractive concept would be the potential of placing a WiSE-CRT electrode at the time of surgery for those with existing or predicted pacing indications, thereby avoiding a separate implantation procedure.

## Conclusion and future directions

The ACHD population represents the most complex cohort of patients who need pacing or CRT. There are challenges for any implanting physician, not only technically but also from a preimplantation decision perspective. Randomized trials are extremely difficult to perform in this population, and thus clinical outcome data are derived primarily from observational studies and registries, which are vulnerable to bias. There is considerable heterogeneity in anatomy and comorbidity, but some challenges are highly prevalent, such as issues with transvenous access, presence of mechanical valves, high risk for PICM, and wishing to avoid multiple sternotomies for several epicardial pacing procedures. We believe that leadless ultrasound-based endocardial pacing can become an important tool in the treatment of such patients in light of its small profile, its ability to be implanted by multiple access routes, and the design to safely pace the systemic ventricle. This technology can be used in its current form for certain situations, namely, in CRT. Improved reliability could see its remit expanded to bradycardia pacing indications, where conduction system pacing could potentially be used. Advances in battery technology (eg, rechargeability) as well as longer term data assessing freedom from complications would make the system more attractive as an option for younger patients. Animal studies testing the feasibility of atrial pacing would open this option to a significant number of Fontan patients with SN dysfunction. In a field in which every patient presents a unique test, creative solutions often need to be applied, and WiSE-CRT may provide the multidisciplinary team with an invaluable option for treatment of these highly challenging cases.

## Disclosures

S.B. has an external research grant from Medtronic and is a consultant to Milestone Pharmaceuticals and Alta Thera Pharmaceuticals. C.A.R. receives research funding or consultation fees from Abbott, Medtronic, Boston Scientific, Spectranetics, EBR Systems, and MicroPort.

## References

[1] Auricchio A., Delnoy P.P., Butter C. (2014). Feasibility, safety, and short-term outcome of leadless ultrasound-based endocardial left ventricular resynchronization in heart failure patients: results of the Wireless Stimulation Endocardially for CRT (WiSE-CRT) study. Europace.

[2] Khairy P., Van Hare G.F., Balaji S. (2014). PACES/HRS Expert Consensus Statement on the Recognition and Management of Arrhythmias in Adult Congenital Heart Disease: developed in partnership between the Pediatric and Congenital Electrophysiology Society (PACES) and the Heart Rhythm Society (HRS). Endorsed by the governing bodies of PACES, HRS, the American College of Cardiology (ACC), the American Heart Association (AHA), the European Heart Rhythm Association (EHRA), the Canadian Heart Rhythm Society (CHRS), and the International Society for Adult Congenital Heart Disease (ISACHD). Heart Rhythm.

[3] Chubb H., O’Neill M., Rosenthal E. (2016). Pacing and defibrillators in complex congenital heart disease. Arrhythm Electrophysiol Rev.

[4] Ohuchi H. (2016). Adult patients with Fontan circulation: what we know and how to manage adults with Fontan circulation?. J Cardiol.

[5] Pundi K.N., Johnson J.N., Dearani J.A. (2015). 40-Year follow-up after the Fontan operation: long-term outcomes of 1,052 patients. J Am Coll Cardiol.

[6] Umamaheshwar K.L., Singh A.S., Sivakumar K. (2019). Endocardial transvenous pacing in patients with surgically palliated univentricular hearts: a review on different techniques, problems and management. Indian Pacing Electrophysiol J.

[7] Khairy P., Landzberg M.J., Gatzoulis M.A. (2006). Transvenous pacing leads and systemic thromboemboli in patients with intracardiac shunts. Circulation.

[8] Morgan J.M., Biffi M., Gellér L., ALSYNC Investigators (2016). ALternate Site Cardiac ResYNChronization (ALSYNC): a prospective and multicentre study of left ventricular endocardial pacing for cardiac resynchronization therapy. Eur Heart J.

[9] Emamaullee J., Zaidi A.N., Schiano T. (2020). Fontan-associated liver disease. Circulation.

[10] Rosenthal E., Qureshi S.A., Crick J.C. (1995). Successful long-term ventricular pacing via the coronary sinus after the Fontan operation. Pacing Clin Electrophysiol.

[11] Delling F.N., Hassan Z.K., Piatkowski G. (2016). Tricuspid regurgitation and mortality in patients with transvenous permanent pacemaker leads. Am J Cardiol.

[12] Silvetti M.S., Drago F., Di Carlo D., Placidi S., Brancaccio G., Carotti A. (2013). Cardiac pacing in paediatric patients with congenital heart defects: transvenous or epicardial?. Europace.

[13] McLeod C.J., Jost C.H., Warnes C.A. (2010). Epicardial versus endocardial permanent pacing in adults with congenital heart disease. J Interv Card Electrophysiol.

[14] Bowman H.C., Shannon K.M., Biniwale R., Moore J.P. (2021). Cardiac implantable device outcomes and lead survival in adult congenital heart disease. Int J Cardiol.

[15] Danielsen A.V., Andreasen J.J., Dinesen B. (2023). Chronic post-thoracotomy pain after lung cancer surgery: a prospective study of preoperative risk factors. Scand J Pain.

[16] Elahi M., Dhannapuneni R., Firmin R., Hickey M. (2005). Direct complications of repeat median sternotomy in adults. Asian Cardiovasc Thorac Ann.

[17] Mah D.Y., Prakash A., Porras D., Fynn-Thompson F., DeWitt E.S., Banka P. (2018). Coronary artery compression from epicardial leads: more common than we think. Heart Rhythm.

[18] Ritter P., Duray G.Z., Steinwender C., Micra Transcatheter Pacing Study Group (2015). Early performance of a miniaturized leadless cardiac pacemaker: the Micra Transcatheter Pacing Study. Eur Heart J.

[19] Mitacchione G., Schiavone M., Gasperetti A., Viecca M., Curnis A., Forleo G.B. (2021). Atrioventricular synchronous leadless pacemaker: state of art and broadened indications. Rev Cardiovasc Med.

[20] Rashtian M., Banker R.S., Neuzil P. (2022). Preclinical safety and electrical performance of novel atrial leadless pacemaker with dual-helix fixation. Heart Rhythm.

[21] Shah M.J., Borquez A.A., Cortez D. (2023). Transcatheter leadless pacing in children: a PACES collaborative study in the real-world setting. Circ Arrhythm Electrophysiol.

[22] Bassareo P.P., Walsh K.P. (2022). Micra pacemaker in adult congenital heart disease patients: a case series. J Cardiovasc Electrophysiol.

[23] Calvert P., Yeo C., Rao A., Neequaye S., Mayhew D., Ashrafi R. (2023). Transcarotid implantation of a leadless pacemaker in a patient with Fontan circulation. HeartRhythm Case Rep.

[24] Omdahl P., Eggen M.D., Bonner M.D., Iaizzo P.A., Wika K. (2016). Right ventricular anatomy can accommodate multiple Micra transcatheter pacemakers. Pacing Clin Electrophysiol.

[25] Razeghi O., Strocchi M., Lee A. (2020). Tracking the motion of intracardiac structures aids the development of future leadless pacing systems. J Cardiovasc Electrophysiol.

[26] Wijesuriya N., De Vere F., Mehta V., Niederer S., Rinaldi C.A., Behar J.M. (2023). Leadless pacing: therapy, challenges and novelties. Arrhythm Electrophysiol Rev.

[27] McAlister F.A., Ezekowitz J., Hooton N. (2007). Cardiac resynchronization therapy for patients with left ventricular systolic dysfunction. JAMA.

[28] Young J.B. (2003). Combined cardiac resynchronization and implantable cardioversion defibrillation in advanced chronic heart failure. JAMA.

[29] Janoušek J. (2009). Cardiac resynchronisation in congenital heart disease. Heart.

[30] Chubb H., Bulic A., Mah D. (2022). Impact and modifiers of ventricular pacing in patients with single ventricle circulation. J Am Coll Cardiol.

[31] Moore B.M., Medi C., McGuire M.A., Celermajer D.S., Cordina R.L. (2020). Pacing-associated cardiomyopathy in adult congenital heart disease. Open Heart.

[32] Dubin A.M., Janousek J., Rhee E. (2005). Resynchronization therapy in pediatric and congenital heart disease patients: an international multicenter study. J Am Coll Cardiol.

[33] Cecchin F., Frangini P.A., Brown D.W. (2009). Cardiac resynchronization therapy (and multisite pacing) in pediatrics and congenital heart disease: five years experience in a single institution. J Cardiovasc Electrophysiol.

[34] Miyazaki A., Sakaguchi H., Kagisaki K. (2016). Optimal pacing sites for cardiac resynchronization therapy for patients with a systemic right ventricle with or without a rudimentary left ventricle. Europace.

[35] Wijesuriya N., Elliott M.K., Mehta V. (2022). Leadless left bundle branch area pacing in cardiac resynchronisation therapy: advances, challenges and future directions. Front Physiol.

[36] Sieniewicz B.J., Betts T.R., James S. (2020). Real-world experience of leadless left ventricular endocardial cardiac resynchronization therapy: a multicenter international registry of the WiSE-CRT pacing system. Heart Rhythm.

[37] Reddy V.Y., Miller M.A., Neuzil P. (2017). Cardiac resynchronization therapy with wireless left ventricular endocardial pacing: the SELECT-LV study. J Am Coll Cardiol.

[38] Wijesuriya N., Elliott M.K., Mehta V. (2022). Leadless left ventricular endocardial pacing for cardiac resynchronization therapy: a systematic review and meta-analysis. Heart Rhythm.

[39] Singh J.P., Rinaldi C.A., Sanders P. Leadless ultrasound-based cardiac resynchronization system in heart failure. JAMA Cardiol. http://10.1001/jamacardio.2024.2050.

[40] Echt D.S., Moore D., Cowan M., Valli V.E., Whitehair J.G., Willis N.P. (2010). Chronic implantation of leadless pacing electrodes in the left ventricle of a goat model. Heart Rhythm.

[41] Sieniewicz B.J., Gould J.S., Rimington H.M., Ioannou N., Rinaldi C.A. (2017). Transseptal delivery of a leadless left ventricular endocardial pacing electrode. JACC Clin Electrophysiol.

[42] Elliott M.K., Vergara P., Wijesuriya N. (2022). Feasibility of leadless left ventricular septal pacing with the WiSE-CRT system to target the left bundle branch area: a porcine model and multicenter patient experience. Heart Rhythm.

